# Effects of smoking habit and diagnosed COPD on intensive care unit stay length of surgically treated coronary artery and obstructive peripheral arterial disease patients

**DOI:** 10.1186/1749-8090-10-S1-A227

**Published:** 2015-12-16

**Authors:** Ersin Çelik, İsmail Yürekli, Ufuk Yetkin, Habib Çakir, Köksal Dönmez, Metin Gümüş, Rahika Durusoy, Ali Gürbüz

**Affiliations:** 1 Department of Cardiovascular Surgery, Katip Celebi University Izmir Ataturk Training and Research Hospital, Izmir, Turkey; 2 Department of Public Health, Medical Sciences Faculty, Ege University, Bornova, 35040 Bornova/İzmir, Turkey

## Background/Introduction

Smoking is one of the most important mortality and morbidity factors.

## Aims/Objectives

We examined 868 coronary artery disease and 268 peripheral vascular disease patients who were treated surgically at our clinic between dates January 2007 and December 2010.

## Method

Mean age of 868 coronary artery disease patients were 63,86 ± 11,17 (between 21-91 years) and 268 peripheral arterial disease patients were 65,44 ± 10,37 (between 21-92 years).

## Results

There were 47 COPD patients in 868 patients who underwent surgery for coronary artery disease. In addition, 490 patients were active smoker and 378 were not using tobacco products. Mean Intensive care unit stay for COPD patients was 4,81 day and 3,06 day for patients without COPD. This difference was significant (p < 0.05).There were 31 COPD patients in 268 patients who underwent surgery for peripheral arterial disease. In this group, 172 patients were active smoker and 96 were not smoking. Mean Intensive care unit stay for COPD patients was significantly longer in COPD group (p < 0.05).

## Discussion/Conclusion

We believe that, preoperative smoking cessation and long-term bronchodilator therapy will reduce morbidity rates of our patients in our daily practice.

## Consent

Written informed consent was obtained from the patient for publication of this Case report and any accompanying images. A copy of the written consent is available for review by the Editor-in-Chief of this journal.

